# The Langeland AED project - call for shorter response times (FirstAED)

**DOI:** 10.1186/1757-7241-20-S2-P47

**Published:** 2012-04-16

**Authors:** Finn Lund Henriksen, Henrik Schakow, Axel Brandes, Bo Kaewkongnok, Mogens Lytken Larsen

**Affiliations:** 1 Department af Cardiology, Odense University Hospital, Denmark; 2 The Langeland AED project, Denmark

## Background

The island of Langeland is a part of peripheral Denmark characterized by long ambulance response times and long distances to the nearest hospitals. Cardiopulmonary resuscitation (cpr) is provided by 200 trained lay rescuers. The population has purchased 85 automated external defibrillators (AEDs) which are available around the clock and placed less than two kilometres apart.

Most of the cardiac arrests occur at home and the rescuers are called by their private telephone. The full potential of the Langeland AEDs has not yet been achieved because of too long rescuer response times.

The project is a collaboration between the AED Centre Odense University Hospital, the Region of Southern Denmark, the Langeland AED project and the company FirstAED.

## Methods

Over the next 24 months every rescuer will use an iPhone 4S as a rescuer telephone. The IT-system (FirstAED), based on GPS technology, will immediately find the geographically closest rescuers. The system is not dependent on the rescuers’ residence. At first, the ten closest rescuers are alarmed; if, and as soon as, they do not respond, the next closest batch of ten are alarmed. Three responders participate: Rescuer 1 and 3 hurry to the cardiac arrest and rescuer 2 collects the AED. The FirstAED technology will open all the locked AED cabinets within a radius of one kilometre. During the resuscitation process and before the arrival of the ambulance, the rescuers will receive advice and feedback from the central ambulance service (AMK).

## Results

Over the last two years, the AEDs have been used 7 times at cardiac arrests on Langeland. But only twice did the AED arrive within 3-5 minutes of the initial call: one patient survived with a good result, and the other patient died of an intracranial haemorrhage. In the other five cases the AED arrived within 8-10 minutes of the initial call, one patient survived with severe brain damage and four patients died.

## Conclusion

The Langeland AED project answers a call for shorter response times. The project hopes that the FirstAED GPS technology and the iPhone 4S telephones to the rescuers will shorten the response times to below 5 minutes.

